# Comparative performance study of three Ebola rapid diagnostic tests in Guinea

**DOI:** 10.1186/s12879-020-05339-2

**Published:** 2020-09-15

**Authors:** Zelda Moran, William Rodriguez, Doré Ahmadou, Barré Soropogui, N’ Faly Magassouba, Cassandra Kelly-Cirino, Yanis Ben Amor

**Affiliations:** 1 Center for Sustainable Development, Earth Institute, Columbia University, 475 Riverside Drive, Suite 1040, New York, NY 10025 USA; 2 Draper Richards Kaplan Foundation, Menlo Park, CA USA; 3 Laboratoire des Fièvres Hémorragiques en Guinée, Conakry, Guinea; 4 Foundation for Innovative New Diagnostics (FIND), Geneva, Switzerland

**Keywords:** Laboratory, Diagnostics, Global Health, Ebola, Rapid diagnostic tests

## Abstract

**Background:**

The 2014/15 Ebola outbreak in West Africa resulted in 11,000 deaths and massive strain on local health systems, and the ongoing outbreak in Democratic Republic of Congo has afflicted more than 3000 people. Accurate, rapid Ebola diagnostics suitable for field deployment would enable prompt identification and effective response to future outbreaks, yet remain largely unavailable. The purpose of this study was to assess the accuracy of three novel rapid diagnostic tests (RDTs): an Ebola, an Ebola-Malaria, and a Fever Panel test that includes Ebola, all from a single manufacturer.

**Methods:**

We evaluated the three RDTs in 109 Ebola-positive and 96 Ebola-negative stored serum samples collected during the outbreak in Guinea in 2014/15, and tested by real-time polymerase chain reaction (RT-PCR). Sensitivity, specificity, and overall percent agreement were calculated for each RDT using RT-PCR as a reference standard, stratified by Ct value ranges.

**Results:**

All tests performed with high accuracy on samples with low Ct value (high viral load). The Fever Panel test performed with the highest accuracy, with a sensitivity of 89.9% and specificity of 90.6%. The Ebola and Ebola-Malaria tests performed comparably to each other: sensitivity was 77.1 and 78% respectively, and specificity was 91.7% for the Ebola test and 95.8% for the Ebola-Malaria test.

**Conclusions:**

This study evaluated the accuracy of three novel rapid diagnostic tests for Ebola. The tests may have significant public health relevance, particularly the Fever Panel test, which detects seven pathogens including Ebola. Given limitations to the study resulting from uncertain sample quality, further evaluation is warranted. All tests performed with highest accuracy on samples with low Ct value (high viral load), and the data presented here suggests that these RDTs may be useful for point-of-care diagnosis of cases in the context of an outbreak. Restrictions to their use in non-severe Ebola cases or for longitudinal monitoring, when viral loads are lower, may be appropriate. Highlighting the challenge in developing and evaluating Ebola RDTs, there were concerns regarding sample integrity and reference testing, and there is a need for additional research to validate these assays.

## Background

The Ebola outbreak of 2014–2015 was the largest in history, with an estimated 28,616 cases and 11,310 deaths, primarily across Guinea, Sierra Leone, and Liberia [1]. The true figures are likely higher, particularly among communities with limited access to healthcare, where deaths were never confirmed or recorded. This reflects a death toll larger than any other recorded Ebola outbreak, and represents the most devastating acute public health emergency in modern history [2].

In 2017 and 2018, multiple outbreaks of Zaire Ebolavirus were declared in the Democratic Republic of Congo (DRC), the most recent being the country’s tenth bout of Ebola since 1976 when the virus was discovered [3]. Between August 2018 and September 2019, 2108 deaths among 3157 confirmed cases had been reported [4]. Widespread conflict in the DRC has been a barrier to public health response teams, delaying case detection, contact tracing, and distribution of the rVSV-ZEBOV vaccine to health workers and case contacts [3, 5]. Weaknesses in the health systems of affected countries, socioeconomic factors such as poverty and population density, and cultural practices such as funeral ceremonies were likely contributors to the outbreak’s severity. In addition, Ebola had not previously been a major concern in the region, and symptoms can easily be missed in areas where other febrile diseases such as malaria are rampant [6, 7].

Inadequate diagnostics contribute to significant delays in Ebola patient identification and isolation, giving the virus an opportunity to spread quickly and impacting how quickly and effectively an outbreak is controlled [8, 9]. During the 2014/15 outbreak, the World Health Organization (WHO) recognized the need for a simple, point-of-care (POC) Ebola diagnostic and published target product profiles (TPPs) to encourage test development [10, 11]. Lacking rapid tests, health workers resorted to case definitions based on general symptoms and epidemiologic risk factors. A retrospective analysis using a surveillance dataset of patients in Guinea during 2014/15 revealed that the Ebola case definition used at the time was only 68.9% sensitive and 49.6% specific, placing patients without Ebola at high risk of being hospitalized alongside those who were infected [12].

The only guidance on the use of rapid tests for Ebola from WHO is a brief “Interim Guidance” from March 2015, [13] and the only RDT identified specifically by WHO as appropriate for use, the Corgenix ReEBOV, is no longer on the market [14]. There is a dire need for sustained availability of Ebola RDTs to contain active and future outbreaks. The purpose of this study was to evaluate the accuracy of the Ebola Virus (EBV) components of three Ebola rapid diagnostic tests (RDTs) developed by Chembio Diagnostics (Medford, USA) in response to the urgent need recognized in 2014/2015 [11]: [1] an Ebola-only test [2]; a combination Ebola-Malaria test, and [3] a fever panel test which identifies 7 diseases, including Ebola.

## Methods

### Study site

All three tests were evaluated using frozen serum samples stored at the Viral Hemorrhagic Fever Laboratory (VHFL) in Conakry, Guinea, which was established in 2014 by the Institut Pasteur [15]. Serum samples had been collected from symptomatic patients during the 2014–15 outbreak, and had been tested at the VHFL using the Trombley RT-PCR assay [16], soon after collection. After this initial characterization as Ebola RNA (+) or (−), leftover sera were stored at − 80 °C in the VHFL.

### Diagnostic tests under evaluation

All tests evaluated in the present study use the Dual Path Platform (DPP; Chembio, Medford, USA) technology, which consists of an immunochromatographic test cartridge and a small, battery-operated reflectance reader. The tests are designed for use with EDTA whole blood (venous or capillary), plasma, or serum. The DPP Fever Panel Antigen System (the “Fever Panel Test”) is a multiplex assay detecting *Plasmodium falciparum* (HRP2), pan-Plasmodium (pLDH), and protein antigens specific for Lassa, Pan-Ebola (Zaire, Sudan, Bundibugyo), Marburg, Dengue, Chikungunya, and Zika Viruses. The DPP Ebola Antigen System (the “Ebola Test”) tests for Ebola Zaire antigen, and the DPP Ebola-Malaria Antigen duplex system (the “Ebola-Malaria Test”) detects Ebola Zaire antigen, *Plasmodium falciparum* (HRP2), and pan-Plasmodium (pLDH). All tests are qualitative and detect the VP40 antigen, which is specific to Ebola.

The DPP format uses a sample strip perpendicular to the test strip (in contrast to the classic lateral flow assay format), which delivers sample directly to the test line. Sample (50uL) and buffer are added to a well on the cassette, and migrate to the test site, where Ebola VP40 antigen is captured by the VP40 antibodies on the test line. The buffer dilutes blue and green dye at the test site, thereby indicating that the sample and buffer have migrated properly. Running buffer is then added to the conjugate pad after a 5-min incubation period, solubilizing gold nanoparticles conjugated to mouse VP40 antibodies. The buffer front sends any antigen left on the membrane towards the test line for additional capture, and the gold conjugate binds and accumulates on the test line. Additional buffer follows and washes the membrane for increased contrast and sensitivity.

**Fig. 1:**
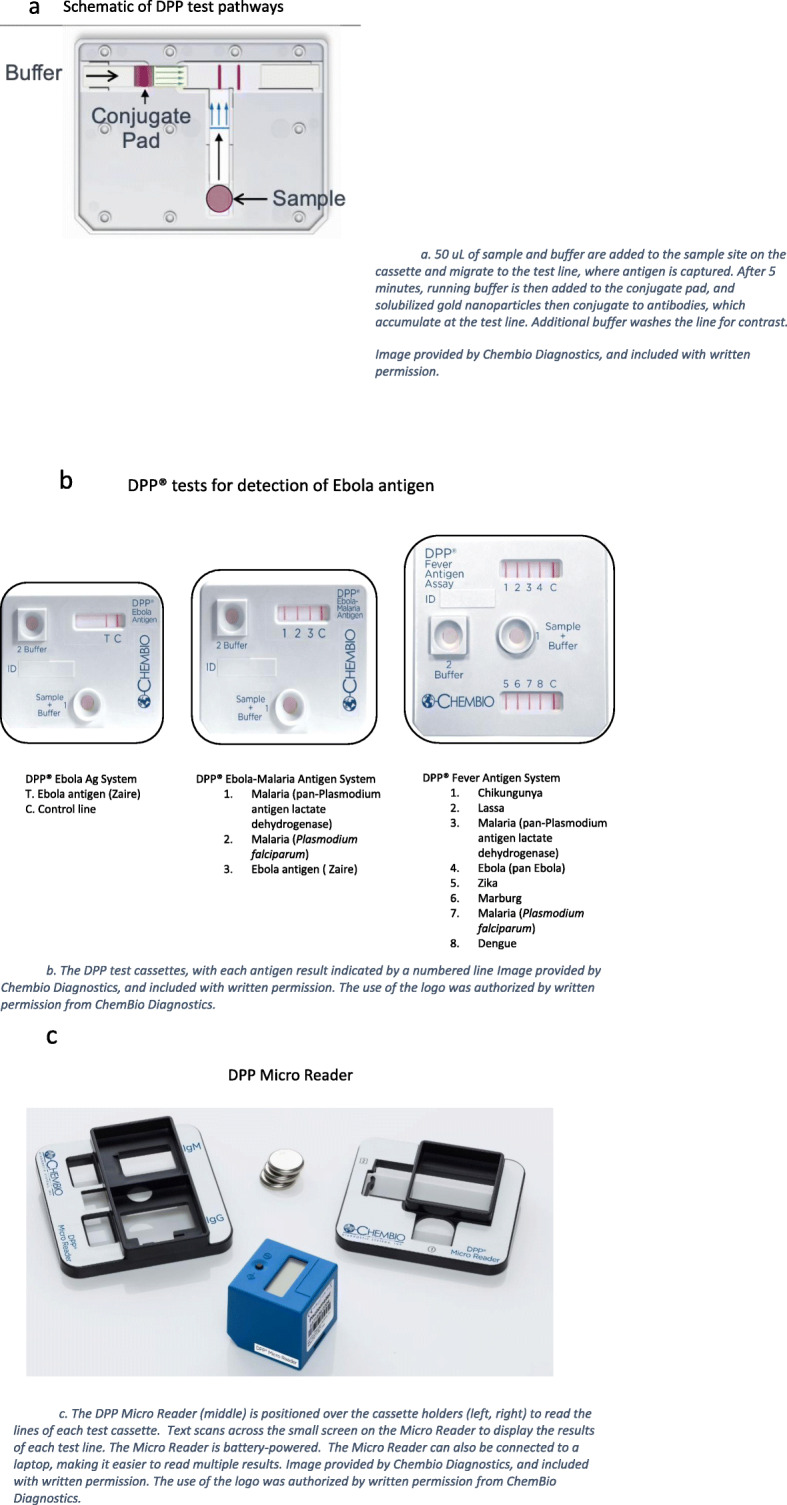
**a** Schematic of DPP test pathways. **a**. 50 uL of sample and buffer are added to the sample site on the cassette and migrate to the test line, where antigen is captured. After 5 min, running buffer is then added to the conjugate pad, and solubilized gold nanoparticles then conjugate to antibodies, which accumulate at the test line. Additional buffer washes the line for contrast. Image provided by Chembio Diagnostics, and included with written permission. **b**. The DPP test cassettes, with each antigen result indicated by a numbered line Image provided by Chembio Diagnostics, and included with written permission. The use of the logo was authorized by written permission from ChemBio Diagnostics. **c**. The DPP Micro Reader (middle) is positioned over the cassette holders (left, right) to read the lines of each test cassette. Text scans across the small screen on the Micro Reader to display the results of each test line. The Micro Reader is battery-powered. The Micro Reader can also be connected to a laptop, making it easier to read multiple results. Image provided by Chembio Diagnostics, and included with written permission. The use of the logo was authorized by written permission from ChemBio Diagnostics

After 10–15 min, a reflectance reader is used to read the test results. This “Micro Reader” analyzes the reflectance of test and control lines to detect VP40 antigen. It interprets the results using assay-specific cut-off values, and reports a reactive Ebola Virus Disease (EVD+), non-reactive (EVD-), or invalid (INV) result as text scrolling through a liquid crystal display (LCD) on the top of the instrument [17]. The DPP tests and Micro Reader are illustrated in Fig. 1a-c. The DPP Ebola assay was previously evaluated in a collaborative study with The National Institute for Biological Standards Control (NIBSC) using samples derived from recombinant VP40 Ebola antigen [18]. All samples of low, medium, or high levels of VP40 tested positive using the Ebola DPP assay. A separate limit of detection study using gamma irradiated serum samples estimated the limit of detection to be 150 ng/ml, corresponding to 7.5 ng/test [17]. Chembio provided technical training and guidance on operating the tests, but was not involved in the funding or execution of this study. As of September 2019, none of the DPP Ebola devices are yet available commercially.

### Sample selection for the study

Ebola-positive and Ebola-negative samples were identified from a database at the VHFL. Samples with sufficient volume of sera were then selected by convenience from the repository based on inclusion criteria. Samples from all patients of any sex or age who were alive at the time of collection in 2014–2015 were eligible. Positive samples were selected by convenience to reflect a range of pathogen loads by molecular testing, with cycle threshold (Ct) values between 15 and 34 (a lower Ct value representing high pathogen RNA, and a higher Ct value indicating low pathogen RNA). No two samples were selected from the same patient.

### Testing procedure and data management

On-site training to VHFL technicians was provided by the study team, according to manufacturer’s instructions, in January 2017, and included use of the diagnostic tests, the Micro Reader, and data management using a tablet. VHFL laboratory technicians performing the tests were blinded to the original RT-PCR result from 2014/15. All testing was completed between February and December 2017. Results were recorded in a Microsoft Excel data form. Micro Reader results were also exported to Excel. While the manufacturer had no role in the collection or analysis of the data, the data obtained from this study have been used by the manufacturer to support a regulatory submission.

### Reference testing

The Trombley assay [16] used at the time of sample collection in 2014/15 was considered the reference standard. Sample use was restricted due to limited volume of sera, so it was not possible to retest all samples with RT-PCR prior to starting the study to confirm integrity, an important limitation to this study. Technicians performing all tests were blinded to sample status.

Sensitivity, Specificity and Accuracy were calculated with 95% confidence intervals for all three assays in reference to the Trombley PCR, including stratification by Ct value (Tables 2 and 3). Positive and negative predictive values were not included in this analysis given the potential biases associated with such a select dataset.

Since the samples had been stored for some time when our study was conducted, a portion were re-tested using a second PCR in order to confirm the integrity of the samples. The Trombley PCR was unavailable, so the RealStar Filovirus Screen RT-PCR Kit 1.0 (altona Diagnostics, Hamburg, Germany), hereinafter “altona” [19] was used instead. Since only convenience sampling was possible for this stage of testing, the results were not considered in formal analyses, but only to shed light on several limitations to data interpretation. These PCR results were further limited by the fact that, in 2017—after investigative rapid testing was completed, but before testing using the altona RT-PCR was completed,—, all samples were transported to the United States, by agreement between VHFL and the US Centers for Disease Control (CDC), and subjected to 5.10^− 6^ rads of gamma irradiation before being shipped back to Guinea. This was unanticipated and not related to the study protocol; however, the gamma irradiation and double shipment may have impacted sample integrity, resulting in variability between the Trombley and altona RT-PCR results.

### Ethics statement

This study was approved as non-human subjects research by Columbia University Institutional Review Board with protocol number AAAR1524(M00Y01), as well as by the National Ethics Committee for Health Research (Comité National D’Ethique Pour La Recherche en Santé – CNERS) of Guinea. All samples were anonymized prior to analysis and no identifying information was used for this study.

## Results

### Study demographic

Two hundred five samples—109 positive and 96 negative samples—were included in the original dataset, of which 55 (50%) had Ct values between 15 and 25, 29 (27%) had Ct values between 26 and 30, and 25 (23%) had Ct values between 31 and 34 (mean: 25.1; range: 16.5–33.8). The study population was 55% (110/205) male, with a mean age of 39 years, and an age range from 3 to 90 years. Samples were selected by convenience based on inclusion criteria and volume of sera.

### Comparison of rapid test results to PCR

Each of the 205 samples was tested using the DPP Ebola, Ebola-Malaria, and the Fever Panel assays, and the result of each was compared to the original PCR result from the time of sample collection in 2014/15. The results are presented for each assay in Tables 1a-c, in the form of 2 × 2 tables.

**Table 1:** Results of Chembio Rapid Diagnostic Tests compared to PCR

Fever Panel (detects PAN)			Ebola (detects Zaire)			Ebola-Malaria (detects Zaire)		
	PCR+	PCR-		PCR+	PCR-		PCR+	PCR-
T+	98	9	T+	84	8	T+	85	4
T-	11	87	T-	25	88	T-	24	92
Total	205		205			205		

When comparing the rapid test result to the original PCR, 20 samples (9.76%) were discordant when using the Fever Panel test, 33 samples (16.1%) were discordant when using the Ebola test, and 28 samples (13.7%) were discordant when using the Ebola-Malaria test. This resulted in the highest sensitivity being 89.91% (95% CI 82.3–94.6%) when using the Fever Panel test, and the lowest being 77.06% sensitivity with the Ebola test. Specificity was highest with the Ebola-Malaria test (95.83%), and lowest with the Fever Panel test (90.63%), see Table 2.

**Table 2:** Accuracy of DPP tests in comparison to Trombley PCR (2014–2015)

Results of DPP Assays Relative to PCR (***n*** = 205) All values are percentages with 95% confidence intervals			
	Fever Panel [95%CI]	Ebola [95%CI]	Ebola Malaria [95%CI]
**Sensitivity**	89.91 (82.3–94.6)	77.06 (67.8–84.3)	77.98 (68.8–85.1)
**Specificity**	90.63 (82.5–95.4)	91.67 (83.8–96.1)	95.83 (89.1–98.7)
**% Discordant (n)**	9.76 (20)	16.1 (33)	13.7 (28)
**Accuracy**	90.24 (85.33–93.94)	83.9 (78.14–88.65)	86.3 (80.87–90.73)

The measures of accuracy were calculated as follows:

**Table:** 

**Measure**	**Formula**
Sensitivity	(TP/(TP+ FN)) * 100%)
Specificity	(TN/(FP+TN)) * 100%)
Accuracy	((TP+TN)/(TP+TN+FP+FN))*100%)
TP = # True Positives; FP = # False Positives; TN=# True Negatives; FN=# False Negatives

### Discordant samples and retesting

Of the 205 samples, 43 (21%) had at least one rapid test result (Fever Panel, Ebola, or Ebola Malaria) that was discordant with the Trombley PCR, with Ct values ranging between 20 and 34. Of the 43 samples with discordant results, 24 (56%) were retested with altona PCR; the remaining 19 samples were unavailable for retesting due to insufficient sample volume. The majority of the retested samples (*n* = 13, or 54%) were Ebola-positive in 2014/15 but retested as negative with the altona PCR, suggesting the possibility of sample degradation or impact from irradiation. Nine samples (37.5%) maintained original Ebola status across both RT-PCR tests, and 6 (67%) of these were positive samples. All 6 of these positive samples had a higher Ct value in 2017 compared to 2014, suggestive of partial sample degradation, or difference in the performance of the two RT-PCR methods.

## Discussion

The aim of this study was to evaluate the performance of a set of novel Ebola RDTs with serum samples from Guinea collected during the 2014/15 outbreak. The DPP Ebola, DPP Ebola-Malaria, and DPP Fever Panel Antigen System RDTs displayed sensitivities of 77.1–89.9% in reference to a Trombley RT-PCR performed at the time of sample collection in 2014/15, with higher sensitivity when using samples with low Ct value (high viral load). The Fever Panel assay showed the highest diagnostic accuracy of the three tests. Generally, the high accuracy of the Ebola test in the Fever Panel may be particularly useful when differentiating Ebola from infection with other pathogens, such as malaria parasites [20]. A follow-up study under more controlled conditions and with reliable baseline RT-PCR results for all samples would help to clarify this issue.

The Ebola component of the Ebola and Ebola-Malaria DPP tests performed with lower sensitivity in this study, particularly among samples with low viral loads (high Ct values), see Table 3. This may be because the Fever Panel test contained additional antibodies for pan-Ebola selectivity (Ebola Zaire, Sudan, and Bundibugyo), while the other two assays are optimized only for Ebola Zaire (personal communication, Chembio). If this holds true in other datasets, the Ebola and Ebola-Malaria tests might be suitable for symptomatic patients with high viral loads, but might not be recommended for contact tracing, for convalescent patients with lower viral burden, or in cases where the outbreak is not confirmed to be Ebola Zaire [21].

**Table 3:** Results (sensitivity) by Ct value range

	Sensitivity by Ct value Range: (n; 95% CI) All values are percentages with 95% confidence intervals		
Ct value range	Fever Panel	Ebola	Ebola-Malaria
**15–25**	96.4% (*n* = 55; 86.4–99.4%)	89.1% (*n* = 55; 77.1–95.5%)	89.1% (*n* = 55; 77.1–95.5%)
**25.01–30**	92.9% (*n* = 28; 75.0–98.8%)	72.41% (*n* = 29; 52.5–86.5%)	75.86% (*n* = 29; 56.0–89.0%)
**30.01–34**	76.0% (*n* = 25; 54.5–89.8%)	56.00% (*n* = 25; 35.27–74.98%)	56.00% (*n* = 25; 35.27–74.98%)

The Ebola RDT TPP published by the World Health Organization describes a desired sensitivity of > 98%, and an acceptable sensitivity of > 95% [11]. In this investigation, all DPP assays fell short of meeting these cutoffs, particularly when used on samples with high Ct value (low viral load).

Nonetheless, as of September 2019, no RDT meeting the specifications suggested by the WHO are commercially available. Two Ebola RDTs have had emergency use assessment and listing (EUAL) from the WHO: the OraQuick Ebola Rapid Antigen Test Kit (Orasure Technologies, USA), and the ReEBOV Antigen Rapid Test Kit (Corgenix, USA) [22]. The OraQuick has a reported sensitivity of 94.1%, (95% CI 83.8–98.8%) and specificity of 100% (98.11–100%) in patients from Sierra Leone [23]. The ReEBOV Antigen Rapid Test had a reported sensitivity of 91.8% (95% CI 84.5–96.8%) in one study [23], and sensitivity and specificity of 100 and 92.2% among 28 high viral load samples from Sierra Leone [24, 25]. However, an initial Emergency Use Authorization (EUA) for the ReEBOV test was revoked by the FDA in 2018 when sufficient performance data could not be replicated by a new manufacturer that had acquired the ReEBOV test. Thus, only the OraQuick test has data supporting an emergency authorization for use in Ebola control.

While the tests evaluated in this study did not meet desired cutoffs for all Ct value ranges, this study clearly highlights the urgent need for a head-to-head comparative study using standardized samples to accurately assess the performance characteristics of the currently available Ebola RDTs. Overall, the tests evaluated in this study met many of the other characteristics outlined in the TPP [11], including time to result (less than 30 min), number of steps required by operator (three), and test stability during storage. The Micro Reader is small and portable, and reading is made easier by using it with a tablet to display the results of multiplex assays.

### Limitations

While representing a unique evaluation of Ebola RDTs, we note several limitations to this study. Overall, the samples had unclear storage history, which was the reason behind using two different RT-PCR reference standards, selected based on availability in 2014 and 2017 [16, 19]. The quality of the samples available in 2017 was uncertain, and samples may have been exposed to freeze-thaw cycles prior to the study, which could have resulted in antigen degradation. This would mean that a positive sample in 2014/15 could have been negative on repeat testing in 2017. However, varying sensitivities of the two RT-PCR methods themselves make these results difficult to interpret, and since the altona PCR could only be applied to a subset of samples, these test results were not considered when calculating measures of accuracy. In addition, all Ebola study samples were sent to the United States for gamma irradiation before being returned to Guinea. The double shipment and gamma irradiation of the samples took place before confirmatory RT-PCR testing, but after testing with the DPP RDTs as part of this research study, and may have additionally contributed to sample degradation. While gamma rays are often used as a safe decontamination method in external quality assurance studies [26], it has also been shown to impact the sensitivity of RT-PCR, possibly by causing nicks in the viral genome [27, 28], and therefore could result in false-negative results from altona. The extent of this impact is unknown.

While multiple factors resulted in important limitations to the interpretation of these results, the hurdles (both expected and unexpected) faced during study execution would likely apply to many, if not most other attempts to evaluate an Ebola rapid diagnostic using banked samples. The results of the altona PCR analysis indicated that stored samples had likely degraded over time, where originally low positives became negative, or that the irradiation had negatively impacted sample quality. Scarce data on the impact of gamma irradiation on viral RNA means that a PCR may be negative, while an antigen-based test could still be positive after irradiation. Given the unknown impact of storage conditions and irradiation on these samples, it is unlikely that another study using these samples could result in more a definitive evaluation of a new diagnostic. The recent withdrawal of the ReEBOV RDT from the market underscores this challenge, and the dearth of Ebola RDTs on the market is perhaps a reflection of the dilemmas associated with safe and high-quality field evaluation for Ebola tests. It is clear that in order to support development and evaluation of new, high quality diagnostics, careful sample processing, characterization, and storage should be prioritized for research purposes whenever possible.

## Conclusion

We evaluated the sensitivity and specificity of the Ebola component of three novel rapid diagnostic tests: one for Ebola alone, one for Ebola and Malaria, and one Fever Panel containing 7 diseases including Ebola. The tests performed with mixed results, the Fever Panel being the most accurate of the three, and better results were observed when using samples with high pathogen load. Given the potential public health impact of rapid diagnostic tests for Ebola, and in particular the differential diagnosis implied by the Fever Panel, further research with high quality samples under more controlled conditions is warranted.

## Data Availability

The datasets used and/or analyzed during the current study are available from the corresponding author on reasonable request.

## Abbreviations

CDC: Centers for Disease Control
Ct: Cycle threshold
DPP: Dual Path Platform
DRC: Democratic Republic of Congo
EBV: Ebola Virus
EUA: Emergency Use Authorization
EUAL: Emergency Use Assessment and Listing
EVD: Ebola Virus Disease
NIBSC: National Institute for Biological Standards Control
PCR: Polymerase Chain Reaction
POC: Point of Care
RDT: Rapid Diagnostic Test
RT-PCR: Real-Time Polymerase Chain Reaction
TPP: Target Product Profiles
VHFL: Viral Hemorrhagic Fever Laboratory
WHO: World Health Organization
